# Supplementary material for: Classification Model for Diabetic Foot, Necrotizing Fasciitis, and Osteomyelitis
Source: Biology (Basel). 2022 Sep 3;11(9):1310. doi: 10.3390/biology11091310 (PMC9495746; doi:10.3390/biology11091310)
Supplement: Supplementary file 1 [file biology-11-01310-s001.zip › biology-1848211-supplementary.pdf]

Supplementary Materials

Classification model for Diabetic foot, Necrotizing fasciitis, and Osteomyelitis

Jiye Kim[1,†], Gilsung Yoo[2,†], Taesic Lee[3,4,6], Jeong Ho Kim[1], Dong Min Seo[5], Juwon Kim[2, 6,*]

Table S1. References in the literature-based search for variables

Reference title	Author	Year	PMID	Feature*
The LRINEC (Laboratory Risk Indicator for Necrotizing Fasciitis) score: a tool for distinguishing necrotizing fasciitis from other soft tissue infections **[1]**	Wong CH, et al.	2004	15241098	CRP<br>WBC<br>Hb<br>Na<br>Cr<br>Glucose
Early diagnosis of necrotizing fasciitis [2]	Goh T, et al.	2014	24338771	Presence of bullae<br>Gas on plain X-ray
Risk factors associated with necrotizing fasciitis of the lower limbs: A multicenter case-control study **[3]**	Pitché P, et al.	2021	33558036	Obesity<br>Nicotine addiction<br>Use of NSAIDs<br>Voluntary cosmetic depigmentation
Hepatitis C viral infection as an associated risk factor for necrotizing fasciitis **[4]**	Scher D, et al.	2012	22495851	HCV infection
Modified Laboratory Risk Indicator for Necrotizing Fasciitis (m-LRINEC) Score System in Diagnosing Necrotizing Fasciitis: A Nested Case-Control Study **[5]**	Wu H, et al.	2021	34113137	Kidney diseae
Usefulness of serum procalcitonin for necrotizing fasciitis as an early diagnostic tool **[6]**	Kishino T, et al.	2021	33454216	Procalcitonin
The infected diabetic foot: Can serum biomarkers predict osteomyelitis after hospital discharge for diabetic foot	Crisologo PA, et al.	2020	32698253	ESR<br>IL-8

infections? **[7]**				IL-6<br>MCP-1
Utility of modified Laboratory Risk Indicator for Necrotizing Fasciitis (MLRINEC) score in distinguishing necrotizing from non-necrotizing soft tissue infections **[8]**	Wu PH, et al.	2021	34039397	Lactate<br>Liver disease
Clinical, microbiological and inflammatory markers of severe diabetic foot infections **[9]**	Aragón-Sánchez J, et al.	2021	34270826	Skin necrosis<br>Albumin<br>NLR
*Laboratory indicators for early detection and surgical treatment of vibrio necrotizing fasciitis***[10]**	Tsai YH, et al.	2010	20232179	Platelet<br>Band form leukocyte
*The SIARI Score: A Novel Decision Support Tool Outperforms LRINEC Score in Necrotizing Fasciiti***[11]***s*	Benjamin I Cribb, et al.	2019	31214830	Site other than lower limb<br>History of immunosuppression<br>Age
*Accurate and quick predictor of necrotizing soft tissue infection: Usefulness of the LRINEC score and NSTI assessment score***[12]**	Harasawa T, et al.	2020	31711831	Mean arterial pressure
*Pentraxin-3: A new parameter in predicting the severity of diabetic foot infection?***[13]**	Ozer Balin S, et al.	2019	30767386	Pentraxin-3
Combined clinical and laboratory testing improves diagnostic accuracy for osteomyelitis in the diabetic foot [14]	Fleischer AE, et al.	2009	19110158	Ulcer depth > 3mm

*Once a feature was introduced in reference, duplicated one in other reference was omitted.

Reference

1. Wong, C.H.; Khin, L.W.; Heng, K.S.; Tan, K.C.; Low, C.O. The LRINEC (Laboratory Risk Indicator for Necrotizing Fasciitis) score: a tool for distinguishing necrotizing fasciitis from other soft tissue infections. *Crit Care Med* **2004**, *32*, 1535-1541. doi:10.1097/01.ccm.0000129486.35458.7d.
2. Goh, T.; Goh, L.G.; Ang, C.H.; Wong, C.H. Early diagnosis of necrotizing fasciitis. *Br J Surg* **2014**, *101*, e119-125. doi:10.1002/bjs.9371.

3. Pitché, P.; Diata, A.B.; Faye, O.; Tounkara, T.M.; Niamba, P.; Mouhari-Toure, A.; Ly, F.; Soumah, M.M.; Some-Korsaga, N.; Akakpo, A.S.; et al. Risk factors associated with necrotizing fasciitis of the lower limbs: A multicenter case-control study. *Ann Dermatol Venereol* **2021**, *148*, 161-164. doi:10.1016/j.annder.2020.08.056.
4. Scher, D.; Kanlic, E.; Bader, J.; Ortiz, M.; Abdelgawad, A. Hepatitis C viral infection as an associated risk factor for necrotizing fasciitis. *Orthopedics* **2012**, *35*, e510-513. doi:10.3928/01477447-20120327-43.
5. Wu, H.; Liu, S.; Li, C.; Song, Z. Modified Laboratory Risk Indicator for Necrotizing Fasciitis (m-LRINEC) Score System in Diagnosing Necrotizing Fasciitis: A Nested Case-Control Study. *Infect Drug Resist* **2021**, *14*, 2105-2112. doi:10.2147/idr.S313321.
6. Kishino, T.; Asai, N.; Ohashi, W.; Sakanashi, D.; Kato, H.; Shiota, A.; Hagihara, M.; Koizumi, Y.; Yamagishi, Y.; Suematsu, H.; et al. Usefulness of serum procalcitonin for necrotizing fasciitis as an early diagnostic tool. *J Infect Chemother* **2021**, *27*, 787-793. doi:10.1016/j.jiac.2021.01.002.
7. Crisologo, P.A.; Davis, K.E.; Ahn, J.; Farrar, D.; Van Asten, S.; La Fontaine, J.; Lavery, L.A. The infected diabetic foot: Can serum biomarkers predict osteomyelitis after hospital discharge for diabetic foot infections? *Wound Repair Regen* **2020**, *28*, 617-622. doi:10.1111/wrr.12836.
8. Wu, P.H.; Wu, K.H.; Hsiao, C.T.; Wu, S.R.; Chang, C.P. Utility of modified Laboratory Risk Indicator for Necrotizing Fasciitis (MLRINEC) score in distinguishing necrotizing from non-necrotizing soft tissue infections. *World J Emerg Surg* **2021**, *16*, 26. doi:10.1186/s13017-021-00373-0.
9. Aragón-Sánchez, J.; Víquez-Molina, G.; López-Valverde, M.E.; Aragón-Hernández, J.; Rojas-Bonilla, J.M.; Murillo-Vargas, C. Clinical, microbiological and inflammatory markers of severe diabetic foot infections. *Diabet Med* **2021**, *38*, e14648. doi:10.1111/dme.14648.
10. Tsai, Y.H.; Hsu, R.W.; Huang, K.C.; Huang, T.J. Laboratory indicators for early detection and surgical treatment of vibrio necrotizing fasciitis. *Clin Orthop Relat Res* **2010**, *468*, 2230-2237. doi:10.1007/s11999-010-1311-y.
11. Cribb, B.I.; Wang, M.T.M.; Kulasegaran, S.; Gamble, G.D.; MacCormick, A.D. The SIARI Score: A Novel Decision Support Tool Outperforms LRINEC Score in Necrotizing Fasciitis. *World J Surg* **2019**, *43*, 2393-2400. doi:10.1007/s00268-019-05061-4.
12. Harasawa, T.; Kawai-Kowase, K.; Tamura, J.; Nakamura, M. Accurate and quick predictor of necrotizing soft tissue infection: Usefulness of the LRINEC score and NSTI assessment score. *J Infect Chemother* **2020**, *26*, 331-334. doi:10.1016/j.jiac.2019.10.007.
13. Ozer Balin, S.; Sagmak Tartar, A.; Uğur, K.; Kilinç, F.; Telo, S.; Bal, A.; Balin, M.; Akbulut, A. Pentraxin-3: A new parameter in predicting the severity of diabetic foot infection? *Int Wound J* **2019**, *16*, 659-664. doi:10.1111/iwj.13075.
14. Fleischer, A.E.; Didyk, A.A.; Woods, J.B.; Burns, S.E.; Wrobel, J.S.; Armstrong, D.G. Combined clinical and laboratory testing improves diagnostic accuracy for osteomyelitis in the diabetic foot. *J Foot Ankle Surg* **2009**, *48*, 39-46. doi:10.1053/j.jfas.2008.09.003.
